# Retraction: Antitumor Activity of Di-*n*-Butyl-(2,6-Difluorobenzohydroxamato)Tin(IV) against Human Gastric Carcinoma SGC-7901 Cells *via* G2/M Cell Cycle Arrest and Cell Apoptosis

**DOI:** 10.1371/journal.pone.0312145

**Published:** 2024-10-10

**Authors:** The PLOS ONE Editors

After this article [1] was published, concerns were raised regarding results presented in Figs 5, 6, 7, and 8, classification of certain cell lines, and reporting of the tumor study methodology.

Specifically:

- In the 0 µmol/L and 5.0 µmol/L panels in Figs 5A and 5B, areas around some cells appear to be discontinuous with the adjacent background, and some cells appear similar to each other.
- In Fig 6C, several bands appear similar to each other:
  ○ lanes 2–3 of the Bax Concentration panel appear similar to lanes 3–4 of the Bax Time panel
  ○ for proteins Bcl-2, Caspase 3, Cyt C, and β-actin, lanes 1–2 of the Concentration panels appear similar to lanes 1–2 of the corresponding Time panels
  ○ lane 3 of the Bcl-2 Concentration panel appears similar to lane 4 of the Bcl-2 Time panel
  ○ lane 3 of the Cyt C Concentration panel appears similar to lane 2 of the β-actin Concentration and Time panels

- In panel PCNA 2.5 µmol/L in Fig 7A, when levels are adjusted to visualize the background, there appear to be vertical and horizontal discontinuities in the lower left-hand region. The corresponding author LY provided the underlying image for the PCNA 2.5 µmol/L panel in Fig 7A, but this did not resolve the concerns.
- In Fig 8B, there appear to be vertical discontinuities in the backgrounds between some lanes and several bands appear similar to each other:
  ○ lanes 1–2 of the Concentration panels appear similar to lanes 1–2 of the corresponding Time panels for all proteins
  ○ lane 4 of the Cyclin B1 Concentration panel appears similar to lane 3 of the Cyclin B1 Time panel
  ○ lane 4 of the β-actin Concentration panel appears similar to lane 3 of the β-actin Time panel

The corresponding author LY stated that the original underlying data for Figs 5A, 5B, 6C, and 8B are no longer available, but provided images from parallel or subsequent replication experiments that did not resolve the concerns.

Concerns have also been raised about potential contamination or misidentification of several cell lines used in this study, including Hep G2 [2], SHSY5Y [3], SGC-7901 [4, 5], and HL-7702 [4]. Upon editorial follow-up, no cell line authentication data were provided. Additionally, the origin of the cell line named EC and described in [1] as human embryonal carcinoma could not be confirmed.

Additional concerns were raised about whether the mouse tumor experiments were conducted according to internationally accepted standards. Upon editorial follow-up, no additional information about humane endpoint criteria or health monitoring was provided and the individual-level tumor size data have not been provided; as such this issue could not be resolved.

In light of the above concerns, the *PLOS ONE* Editors retract this article.

LY did not respond to the editorial decision. LQ and ZJ either did not respond directly or could not be reached.
